# Safety and Efficacy of the Use of Intrathecal Morphine for Spinal Three Column Osteotomy

**DOI:** 10.7759/cureus.1818

**Published:** 2017-11-03

**Authors:** Jason R Audlin, Swamy Kurra, William Lavelle, Richard A Tallarico, Mike H Sun, Nathaniel R Ordway, Elizabeth A Demers Lavelle

**Affiliations:** 1 Department of Orthopedic Surgery, SUNY Upstate Medical University; 2 Department of Anesthesiology, SUNY Upstate Medical University

**Keywords:** intrathecal morphine, postoperative pain management, efficacy, safety, side effects, spinal deformity surgery

## Abstract

Introduction

The use of intrathecal morphine has the potential to help alleviate the pain that patients experience undergoing spinal surgeries. Complications can cause immobilization, which can lead to vascular thrombosis and ileus. Studies have shown epidural analgesia significantly lowered postoperative pain scores in scoliosis surgeries. Intrathecal anesthesia has been shown to have good pain control over the initial 24-hour postoperative period.

Purpose

Determine if intrathecal morphine would reduce postoperative pain with minimal side effects.

Methods

The surgical case logs from three spinal deformity surgeons from a single academic medical center were reviewed retrospectively. This included cases where more than five levels of fusion occurred and surgery involved an osteotomy. The records of 17 patients were queried, and patient and surgical data were collected. The patients were divided into two groups: eight patients were administered intrathecal morphine and nine patients received no morphine. Postoperative pain scores were obtained hourly over the initial 24 hours postoperatively by nurses trained to obtain pain scores from the Numeric Pain Rating Scale. In addition, the rates of any noted side effects were recorded. Analysis of variance (ANOVA) and Fisher’s exact tests were used to calculate any statistical significance with p < 0.05 considered to be significant.

Results

The maximum and total 24-hour postoperative pain scores had a mean of 5.6 (standard deviation = 4.2; p = 0.4266) and 69.3 (standard deviation = 57.8; p = 0.9189), respectively, for patients administered intrathecal morphine. The patients who did not receive intrathecal morphine had total pain scores of 3.9 (standard deviation = 4.5) and 65.7 (standard deviation = 79.7), respectively. Though the results were not statistically significant, there was a potential trend toward decreased in pain mean scores in the first 10 hours for the intrathecal morphine group. There was no statistical difference in the rate of side effects between patients.

Conclusions

The use of intrathecal morphine did not significantly appear to reduce postoperative pain in patients when compared to intravenous or oral narcotics. There was a potential trend in a reduction in postoperative pain during the first 10 hours postoperatively, but this did not reach a statistically significant value and did not hold up after the first 10 hours postoperatively. However, it was noted that intrathecal morphine was safe to use in postoperative spinal deformity surgery as no statistical significance in side effects was noted.

## Introduction

Patients who undergo spinal deformity surgery often have great difficulty in recovery in part due to the high intensity of pain reported over the course of their recovery. This pain often results in difficulty in mobilization for the patient who then is put at higher risk for developing serious complications, such as venous thrombosis and ileus, and thus, also delaying rehabilitation. Methods that would reduce the pain reported by patients who have undergone spinal deformity surgery would, therefore, increase the quality of their postoperative care and potentially aid in progressing their rehabilitation and recovery. Previous studies have reported the use of epidural analgesia significantly lowered the reported pain scores of patients who underwent surgeries to correct diagnosed scoliosis [1]. Furthermore, other studies have shown the potential of intrathecal anesthesia as pain relief for treatment of cancers and chronic pain, as well as postoperative pain relief following spinal surgeries [2-12]. The use of intrathecal morphine could potentially be useful in controlling the pain experienced by spinal surgery patients postoperatively; however, there have been reported side effects, which include pruritis, urinary retention, nausea and vomiting, and the most serious of all, respiratory depression [11]. The purpose of this study was to examine the pain relief of patients undergoing spinal deformity surgery who were given intrathecal morphine in comparison to patients who underwent spinal deformity surgery without being given intrathecal morphine for pain relief.

## Materials and methods

With Institutional Review Board approval from our institution, the medical records of 17 consecutive adult patients who received spinal instrumentation involving more than five levels with osteotomy were reviewed. These patients were under the care of three fellowship-trained spinal deformity surgeons from a single academic medical center from October 2008 to October 2012. The data that was reviewed and collected included patient demographic data, gender, age at the time of surgery, and surgical data. The surgical data collected included the number of levels of fusion, the postoperative pain scores, and the rates of any noted side effects, including pruritus, nausea, nausea medication use, vomiting, ileus, constipation, urinary retention, change in the neurologic examination, and rates of infection. The postoperative pain scores were obtained hourly, recorded, and measured by nurses trained in obtaining pain scores. The Numeric Pain Rating Scale (0-10) was used to calculate the pain scores: 0 = no pain, 1-3 = mild, 4-6 = moderate, and 7-10 = severe [13].

Patients were categorized based on intrathecal morphine (IM) use. A single dose of morphine (0.6 mg) was administered using an intrathecal catheter, which was placed during surgery in the lumbar region to control postoperative pain in the intrathecal morphine group. The protocol for the management of postoperative pain in patients with and without IM group was same, i.e, continuous intravenous patient-controlled anesthesia (IV PCA); fentanyl was used in IV PCA. From the recorded 24-hour pain scores, we calculated postoperative Day 1 total pain scores by adding all pain scores for those who received intrathecal morphine and those who did not receive intrathecal morphine separately.

We compared the postoperative Day 1 total pain scores between patients (with and without morphine) using analysis of variance (ANOVA) to see whether there was any difference in those pain scores. Summary statistics and ANOVA were performed with standard statistical testing, depending on data type and distribution. Fisher’s exact test was used to compare categorical data, whereas ANOVA tests were used to make comparisons on the basis of means. SAS (Statistical Analysis System) 9.4 (SAS Institute, Inc., Cary, NC) was used to perform the statistical analysis and Microsoft Excel® (Microsoft Corp., Redmond, WA) was used to plot the graph. Statistical significance was defined by a probability value of < 0.05.

## Results

Of the 17 total patients included in this study, eight patients who had spinal deformity surgery with more than five levels of fusion with instrumentation and involving an osteotomy received standard intrathecal morphine (IM) dosage, while the remaining nine patients did not receive intrathecal morphine (NIM). Patient demographics are summarized in Table 1. 

**Table 1:** Summary of Patient Demographics F: female; M: male; N: no; Y: yes; IM: intrathecal morphine; NM: neuromonitoring; PSO:  pedicle subtraction osteotomy; ARC: anesthesia-related complication; NMU: nausea medications used; Const: constipation; UR: urinary retention

Sub Id	Age	Gender	IM	IM dose (mg)	Levels fused	NM changes	Osteotomy	ARC
3	76	F	N	--	T11- Pelvis	None	PSO	NMU
4	70	M	Y	0.6	T10-S1	None	PSO	None
5	65	F	N	--	T3-Pelvis	None	PSO	Ileus, Const, UR
6	60	F	Y	0.6	T9-L5	None	PSO	Pruritis, Nausea, NMU
7	64	M	N	--	T10-L4	None	PSO	NMU, Ileus, Const
9	51	M	Y	0.6	T10-Pelvis	None	PSO	Const
11	61	F	Y	0.6	T8-S1	None	PSO	Pruritis, Const
14	52	M	Y	0.6	T8-Pelvis	None	PSO	Const
15	68	F	N	--	T4-Pelvis	None	PSO	UR
19	60	F	N	--	T2-Pelvis	None	PSO	Const
21	70	F	Y	0.6	T5-Pelvis	None	PSO	Nausea, NMU, Vomit, Const
22	66	F	Y	0.6	T8-Pelvis	None	PSO	UNM
24	65	F	N	--	T8-L5	None	PSO	None
18	50	M	Y	0.6	T6-L5	None	PSO	UR
20	58	F	N	--	T3-Pelvis	None	PSO	Nausea, Vomit, Const
23	65	M	N	--	T10-Pelvis	None	PSO	Const
25	67	M	N	--	T10-Pelvis	None	PSO	None

The maximum and total 24-hour postoperative pain scores had a mean of 5.6 (standard deviation = 4.2) and 69.3 (standard deviation = 57.8), respectively, for patients administered intrathecal morphine. This is in comparison to a 24-hour postoperative pain score maximum and total pain score of 3.9 (standard deviation = 4.5) and 65.7 (standard deviation = 79.7), respectively, for patients who did not receive intrathecal morphine. The resulting p-values for each category of data were 0.4266 and 0.9189, respectively (Table 2).

**Table 2:** Pain Scores Within 24 Hours Postop With and Without Intrathecal Morphine (IM) p < 0.05 = statistical significance SD: Standard deviation; IM: intrathecal morphine; N: number

	With IM	Without IM
Maximum pain score within 24 hours postop		
N	8	9
Mean (SD)	5.6 (4.2)	3.9 (4.5)
Median	5.5	3.0
(min, max)	(0, 10)	(0, 10)
p-value	0.4	
Total pain score within 24 hours postop		
N	8	9
Mean (SD)	69.3 (57.8)	65.7 (79.7)
Median	89.0	33.0
(min, max)	(0, 143)	(0, 196)
p-value	0.9	

**Figure 1:**
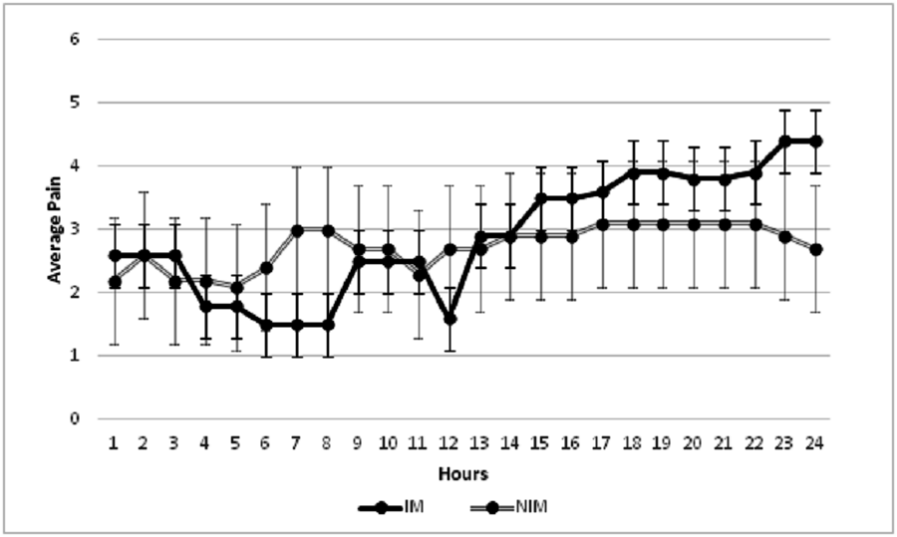
Trend of average pain scores with and without intrathecal morphine (IM) NIM: no intrathecal morphine

While these results were not statistically significant, there was a potential trend toward decreased mean pain scores in the first 10 hours postoperatively for the intrathecal morphine group (Figure 1). However, this did not hold up after 10 hours postoperatively. There was no statistically significant difference between the intrathecal morphine group and the control group (NIM) for any of the noted side effects. These included: constipation (p = 0.3), ileus (p = 1.0), nausea (p = 1.0), pruritis (p = 0.5), urinary retention (p = 1.0), use of nausea medication (p = 1.0), and vomiting (p = 1.0) (Table 3). Respiratory depression was not noticed in patients who received intrathecal morphine. No spinal fluid leaks, postoperative headaches, or new neurological deficits were noted that were associated with the use of intrathecal morphine.

**Table 3:** Complications With and Without Intrathecal Morphine (IM)

Complications	Treatment	Yes/No	Frequency	Percentage (%)	p-value (Fisher’s Exact test)
Constipation	Without IM	No	5	55.6	0.3
		Yes	4	44.4	
	With IM	No	2	25.0	
		Yes	6	75.0	
Ileus	Without IM	No	8	88.9	1.0
		Yes	1	11.1	
	With IM	No	7	87.5	
		Yes	1	12.5	
Nausea	Without IM	No	7	77.8	1.0
		Yes	2	22.2	
	With IM	No	7	87.5	
		Yes	1	12.5	
Pruritis	Without IM	No	8	88.9	0.5
		Yes	1	11.1	
	With IM	No	6	75.0	
		Yes	2	25.0	
Urinary Retention	Without IM	No	7	77.8	1.0
		Yes	2	22.2	
	With IM	No	6	75.0	
		Yes	2	25.0	
Use of nausea medications	Without IM	No	6	66.7	1.0
		Yes	3	33.3	
	With IM	No	5	62.5	
		Yes	3	37.5	
Vomiting	Without IM	No	8	88.9	1.0
		Yes	1	11.1	
	With IM	No	7	87.5	
		Yes	1	12.5	

## Discussion

Overall, this pilot study showed that patients treated with intrathecal morphine did not have a statistically significant reduction in postoperative pain when compared to patients who were not treated with intrathecal morphine. However, there was a potential trend where the patients that received intrathecal morphine showed a reduction in postoperative pain reported in the first 10 hours after surgery. In addition, another trend observed was that patients not receiving intrathecal morphine showed a reduction in reported pain from 13 to 24 hours postoperatively. However, it should be noted that neither of these observed trends achieved statistical significance.        

These observed results stand in contrast to studies in the published literature where intrathecal administration of medications have been able to relieve the pain associated with cancer treatments along with acute and chronic pain management. Deer and Chapple, et al. reported data from the National Outcomes Registry for Lower Back Pain on patients with chronic lower back and leg pain that received intrathecal drug delivery [4]. This data showed that of 136 patients implanted, the pain was reduced by more than 47% for back pain and more than 31% for leg pain [4]. Chambers and Mac Sullivan’s study also showed that 15 patients that received intrathecal morphine for chronic pain relief all reported excellent or good relief for after their therapy with intrathecal morphine [2].

This method of pain management has also shown potential to reduce pain following spinal surgeries. Demircan comments on a study by Ross, et al., where a prospective, double-blind, placebo-controlled study of the use of intrathecal morphine after lumbar spinal surgery, showing that patients receiving intrathecal morphine saw a reduction in parenteral narcotics over the course of their hospitalization as well as a reduction in the mean length of hospitalization when compared to patients who had systemic narcotic administration [5, 14]. Another study from Poblete, et al. also showed that patients that received intrathecal morphine after cervical or thoracic spinal cord tumor surgery reported their highest pain scores immediately postoperatively with a decline at 12 hours postoperatively, along with minimal extra morphine being necessary throughout their hospitalization [10].

Our study also showed that there were no statistically significant observations of noted side effects associated with intrathecal morphine administration. Rathmell, et al. detailed the use of intrathecal drugs in the treatment of acute pain, as well as noting common side effects seen in intrathecal opioid administration, such as pruritus, urinary retention, and nausea and vomiting, along with the most feared side effect of respiratory depression seen in patients receiving large doses of opioids [11]. Gehling and Tryba also performed a meta-analysis to measure the risks and side effects of intrathecal morphine administration that showed patients receiving lower doses of morphine were found to have greater incidences of nausea, vomiting, and pruritus when compared to placebo [15]. Higher doses of morphine showed an increased risk ratio for pruritus, but not for nausea or vomiting, and it was also associated with a greater incidence of respiratory depression [15]. The results of our study, along with the associated data from other published works, add to the conclusion that intrathecal opioid administration is safe to use after spinal deformity surgeries for postoperative pain relief and management. Readers should cautiously review this safety data as a small series such as our study would only have the potential to identify adverse outcomes with a very large effect size.

Limitations of our study include the small sample size, which could reduce the power of the study enough to not be able to detect a statistically significant difference in postoperative complication rates or change in postoperative pain scores. This study should be repeated to include a larger sample size in order to increase the power of the study and allow for statistically significant differences to be detected in the primary and secondary outcomes. Another potential variable that could be investigated in a follow-up study would be the introduction of other local anesthetics in combination with opioids intrathecally for management of pain after spinal deformity surgery. Deer and Caraway, et al. published a study comparing the effects of a combination of bupivacaine and opioids intrathecally with opioids alone for the treatment of pain of spinal origin [3]. Their study revealed the patients receiving the combination therapy showed significantly greater pain reduction, reduction in oral opioid and non-opioid pain medication usage, reduction in doctor, emergency room, or pain clinic visits, and a statistically significant increase in patient satisfaction when compared to the opioids alone group [3]. This data could be warranted to add to our study to see the possible effects this could have for patients.

## Conclusions

In conclusion, the use of intrathecal morphine did not significantly appear to reduce postoperative pain in patients when compared to intravenous or oral narcotics. There did appear to be a potential trend in a reduction in postoperative pain during the first 10 hours postoperatively, but this did not reach a statistically significant value. However, it was noted that intrathecal morphine was safe to use in postoperative spinal deformity surgery as no statistical significance in side effects was noted.

## References

[1] Lavelle ED, Lavelle WF, Goodwin R (2010). Epidural analgesia for postoperative pain control after adolescent spinal fusion procedures which violated the epidural space. J Spinal Disord Tech.

[2] Chambers FA, MacSullivan R (1994). Intrathecal morphine in the treatment of chronic intractable pain. Ir J Med Sci.

[3] Deer TR, Caraway DL, Kim CK (2002). Clinical experience with intrathecal bupivacaine in combination with opioid for the treatment of chronic pain related to failed back surgery syndrome and metastatic cancer pain of the spine. Spine J.

[4] Deer T, Chapple I, Classen A (2004). Intrathecal drug delivery for treatment of chronic low back pain: report from the National Outcomes Registry for Low Back Pain. Pain Med.

[5] Demircan MN (1992). Use of intrathecally administered morphine in the treatment of postoperative pain after lumbar spinal surgery: a prospective, double-blind placebo-controlled study. Neurosurgery.

[6] Gwirtz KH, Young JV, Byers RS (1999). The safety and efficacy of intrathecal opioid analgesia for acute postoperative pain: seven years' experience with 5969 surgical patients at Indian University Hospital. Anesth Analg.

[7] Morgan M (1989). The rational use of intrathecal and extradural opioids. Br J Anaesth.

[8] O'Neill P, Knickenberg C, Bogahalanda S, Booth AE (1985). Use of intrathecal morphine for postoperative pain relief following lumbar spine surgery. J Neurosurg.

[9] Paice JA, Penn RD, Shott S (1996). Intraspinal morphine for chronic pain: a retrospective, multicenter study. J Pain Symptom Manage.

[10] Poblete B, Konrad C, Kothbauer KF (2014). Intrathecal morphine analgesia after cervical and thoracic spinal cord tumor surgery. J Neurosurg Spine.

[11] Rathmell JP, Lair TR, Nauman B (2005). The role of intrathecal drugs in the treatment of acute pain. Anesth Analg.

[12] Winkelmüller M, Winkelmüller W (1996). Long-term effects of continuous intrathecal opioid treatment in chronic pain of nonmalignant etiology. J Neurosurg.

[13] (2017). Rehab Measures: Numeric Pain Rating Scale. http://www.rehabmeasures.org/Lists/RehabMeasures/PrintView.aspx?ID=891.

[14] Ross DA, Drasner K, Weinstein PR (1991). Use of intrathecally administered morphine in the treatment of postoperative pain after lumbar spinal surgery: a prospective, double-blind, placebo-controlled study. Neurosurgery.

[15] Gehling M, Tryba M (2009). Risks and side-effects of intrathecal morphine combined with spinal anaesthesia: a meta-analysis. Anaesthesia.

